# Lichen plan blaschko-linéaire

**DOI:** 10.11604/pamj.2013.15.33.2842

**Published:** 2013-05-25

**Authors:** Kbira El Morabite, Baderddine Hassam

**Affiliations:** 1 Service de dermatologie vénérologie, CHU Ibn Sina, Université Mohamed-V Souissi, Rabat, Maroc

**Keywords:** Lichen blascho-linéaire, lignes de blaschko, lichen pigmentogène, blascho-linear lichen, blaschko lines, pigmentogenic lichen

## Image en médecine

Le lichen blaschko-linéaire constitue une forme rare (0,5 %) du lichen plan dont l'âge moyen de survenue est de 50ans. Il est plus fréquent chez l'homme. Sa disposition linéaire en dehors du phénomène de Koebner serait due au mosaicisme génétique responsable de la présence d'un clone kératinocytaire capable de réagir sous l'effet de facteurs immunogènes, exogènes ou infectieux. Le traitement repose sur la corticothérapie associée à l'émollient. La régression spontanée est possible avec persistance de séquelles pigmentaires. Nous rapportons le cas d'une patiente âgée de 26 ans, sans antécédents pathologiques particuliers, ayant consultée pour des lésions maculo-papuleuses pigmentées prurigineuses évoluant depuis 1an, ces lésions se localisaient le long des lignes de blaschko au niveau de la face postérieure du membre inferieur gauche. L'examen du reste de tégument et des muqueuse était normal. Une biopsie cutanée était réalisée. L'étude histologique était en faveur d'un lichen pigmentogène. La patiente était mise sous émollient et dermocorticoïdes avec une amélioration des lésions et persistance d'une pigmentation séquellaire.

**Figure 1:**
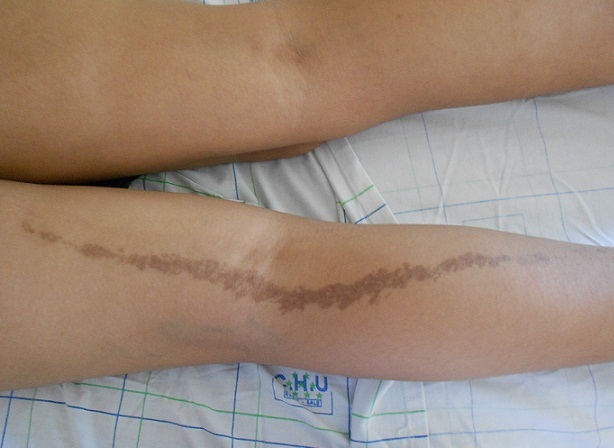
Lésions de lichen plan blaschko-linéaire le long du membre inférieur droit

